# Unconjugated bilirubin induces pyroptosis in cultured rat cortical astrocytes

**DOI:** 10.1186/s12974-018-1064-1

**Published:** 2018-01-22

**Authors:** Jie Feng, Mengwen Li, Qian Wei, Shengjun Li, Sijie Song, Ziyu Hua

**Affiliations:** 1 Department of Neonatology, Children’s Hospital of Chongqing Medical University, Chongqing, 400014 China; 2 Ministry of Education Key Laboratory of Child Development and Disorders, Chongqing, 400014 China; 3 Chongqing Key Laboratory of Translational Medical Research in Cognitive Development and Learning and Memory Disorders, Chongqing, 400014 China; 4 Chongqing International Science and Technology Cooperation Center for Child Development and Disorders, Chongqing, 400014 China; 5 China International Science and Technology Cooperation base of Child development and Critical Disorders, Chongqing, China

**Keywords:** Pyroptosis, Astrocytes, Bilirubin, Neurotoxicity, Inflammation

## Abstract

**Background:**

Bilirubin-induced neurological dysfunction (BIND), a severe complication of extreme neonatal hyperbilirubinemia, could develop into permanent neurodevelopmental impairments. Several studies have demonstrated that inflammation and nerve cell death play important roles in bilirubin-induced neurotoxicity; however, the underlying mechanism remains unidentified.

**Methods:**

The present study was intended to investigate whether pyroptosis, a highly inflammatory form of programmed cell death, participated in the bilirubin-mediated toxicity on cultured rat cortical astrocytes. Further, VX-765, a potent and selective competitive drug, was used to inhibit the activation of caspase-1. The effects of VX-765 on astrocytes treated with bilirubin, including the cell viability, morphological changes of the cell membrane and nucleus, and the production of pro-inflammation cytokines, were observed.

**Results:**

Stimulation of the astrocytes with unconjugated bilirubin (UCB) at the conditions mimicking those of jaundiced newborns significantly increased the activation of caspase-1. Further, caspase-1 activation was inhibited by treatment with VX-765. Compared with UCB-treated astrocytes, the relative cell viability of VX-765-pretreated astrocytes was improved; meanwhile, the formation of plasma membrane pores was prevented, as measured by lactate dehydrogenase release, trypan blue staining, and ethidium bromide (EtBr) uptake. Moreover, DNA fragmentation was partly attenuated and the release of IL-1β and IL-18 was apparently decreased.

**Conclusion:**

Pyroptosis is involved in the process of UCB-induced rat cortical astrocytes’ injury in vitro and may be the missing link of cell death and inflammatory response exacerbating UCB-related neurotoxicity. More importantly, the depression of caspase-1 activation, the core link of pyroptosis, attenuated UCB-induced cellular dysfunction and cytokine release, which might shed light on a new therapeutic approach to BIND.

## Background

Neonatal hyperbilirubinemia remains a common phenomenon among newborns. Although generally harmless, some extremely hyperbilirubinemic neonates may develop bilirubin-induced neurological dysfunction (BIND), including acute bilirubin encephalopathy and permanent neurological sequelae, kernicterus, characterized by cerebral palsy, impaired mental development, and neurological deafness [1]. Despite the dramatically reduced incidence of BIND worldwide, owing to phototherapy and exchange transfusion, this condition remains a severe problem in low-income and middle-income countries, where neonates do not receive timely and prompt intervention [2–4]. Apparently, understanding the mechanisms governing the neurotoxicity of unconjugated bilirubin (UCB) is essential for the development of new strategies of therapeutic intervention. Thus, studies have been performed to increase the understanding of bilirubin neurotoxicity.

Recently, growing evidence has indicated that inflammation plays an important role in the physiological and pathological process of neurological dysfunction induced by UCB [5–7]. Clinical literature suggests that infection increased the risk of bilirubin encephalopathy in newborns [8]. In addition, the treatment of Gunn rats (rat model for Crigler-Najjar syndrome) with the antibiotic minocycline can effectively prevent the loss of cerebellar Purkinje and granule neurons during the neonatal period and attenuate UCB-induced central auditory dysfunction in rat pups [9–11]. Interestingly, the neuroprotective properties of minocycline are demonstrated in part via the suppression of glial (astrocytic/microglial) activation, the inhibition of caspase-1 and caspase-3 expression, and the release of inflammatory mediators [12, 13]. Notably, the activation of glial cells and the secretion of pro-inflammation cytokines are the characteristics of neuroinflammation [14]. Recent studies have demonstrated that elevated levels of UCB activated astrocytes and microglia, as well as gliosis, with a subsequent upregulation of inflammatory markers, such as tumor necrosis factor alpha (TNF-α), interleukin (IL)-1β, and IL-6 [15–18]. In turn, the increased cytokines could further exacerbate nerve cell death and inflammatory responses by activating the MAPK and nuclear factor-kappaB (NF-κB) signaling cascades [17, 19, 20]. Interestingly, blocking pro-inflammatory cytokine production decreased UCB-induced cell death, either by the loss of membrane integrity or by apoptosis [7, 16, 17]. Thus, there may be a link between inflammation responses and nerve cell death caused by UCB, but the precise mechanism remains unclear.

Pyroptosis, a highly inflammatory form of cell death, is mediated by the activation of caspase-1 [21, 22]. Cells undergo pyroptosis, resulting in rapid plasma membrane rupture, osmotic lysis, deoxyribonucleic acid (DNA) fragmentation, and the release of pro-inflammatory cellular contents, which attracts more cells to die [21–23]. Studies have confirmed that this novel form of cell death may play a critical role in the pathological process of nervous system diseases, such as Alzheimer’s disease and epilepsy, which are characterized by nerve cell damage and sterile inflammation [24, 25]. As mentioned above, cell death and inflammatory response are two signature events in the process of BIND. Thus, it is reasonable to postulate that pyroptotic cell death may provide the missing link between nerve cell injury and inflammation caused by UCB. However, to our knowledge, there are few studies examining pyroptotic cell death participated in UCB-mediated neurotoxicity. The aim of the present study was to investigate whether pyroptosis contributes to UCB toxicity on astrocytes, the potent source of inflammatory cytokines and dominant glial cells of the central nervous system (CNS) [14]. Further, VX-765, a potent and selective competitive drug, was used to inhibit the activation of caspase-1. The effects of VX-765 on astrocytes treated with bilirubin were observed, including cell viability, morphological changes of the cell membrane and nucleus, and pro-inflammatory cytokine production.

## Methods

### Materials

#### Reagents

Dulbecco’s modified Eagle’s medium (DMEM) and fetal bovine serum (FBS) were obtained from Gibco (Grand Island, NY, USA). Bilirubin, ethidium bromide (EtBr), Hoechst 33342, 3-(4,5-dimethyl-2-thiazolyl)-2,5-diphenyl-2-*H*-tetrazolium bromide (MTT), human serum albumin (HSA), and trypan blue were purchased from Sigma-Aldrich, Co. (St. Louis, MO, USA). Ethidium homodimer-2 (EthD2) was purchased from Invitrogen (Thermo Fisher Scientific). VX-765 was purchased from Selleck Chemicals (Houston, TX, USA). Anti-caspase-1 antibody (ab1872) and anti-NLR family pyrin domain containing 3 (NLRP3) (ab91413) were purchased from Abcam (Cambridge, UK). Anti-glial fibrillary acidic protein (GFAP) antibody (3670), anti-β-actin (3700), an HRP-conjugated anti-rabbit secondary antibody (7074P2), and an HRP-conjugated anti-mouse secondary antibody (7076P2) were purchased from Cell Signaling Technology. IL-1β enzyme-linked immunosorbent assay (ELISA) kit and IL-18 ELISA kit were from USCN Life Science, Inc. (Wuhan, China). Lactate dehydrogenase (LDH) cytotoxicity detection kit was obtained from Beyotime Biotechnology (Shanghai, China). All other chemicals and reagents were from Beyotime Biotechnology unless otherwise specified.

#### Experimental animals

All animal procedures were approved by the Ethics Committee of Chongqing Medical University (Permit SYXK2007-0016). All experimental Sprague Dawley rats (SPF grade) were obtained from the Animal Experiment Center of Chongqing Medical University. The animal studies were conducted in accordance with the Guide for the Care and Use of Laboratory Animals of the National Institutes of Health. Efforts were made to minimize animal suffering and to reduce the number of animals used.

### Methods

#### Cell culture

Rat primary astrocyte cultures were prepared from the cerebral cortices of 3-day-old Sprague Dawley rats as described by McCarthy and de Vellis [26], with minor modifications. Briefly, following removal of the meninges, blood vessels, and white matter, the cerebral cortex was cut into small pieces and washed in cold DMEM. The pieces were then treated with 0.25% trypsin at 37 °C for 30 min. After neutralizing the trypsin in DMEM containing 10% FBS, the cells were resuspended in DMEM and plated on T75 flasks pre-coated with poly-l-lysine at a density of 2.0 × 10^5^ cells/cm [2]. The cultures were maintained in a humidified atmosphere of 95% air/5% CO_2_ at 37 °C. The media were changed twice weekly. On the 7th–9th day in vitro, the cultured astrocytes were detached by exposure to 0.25% trypsin/0.02% EDTA and subsequently seeded onto poly-d-lysine-coated 6-/24- or 96-well plates. Finally, the astrocytes were used when a confluent monolayer formed with > 95% of the cells stained positively for the astrocytic marker GFAP.

#### Cell treatment

Bilirubin solution preparation was as follows: bilirubin was dissolved in DMSO solution (10 mM) immediately prior to use, and the pH was adjusted to 7.4 with HCl (0.1 M). All experiments with UCB were performed under light protection to avoid photodegradation. VX-765 was dissolved in DMSO solution (50 mM) and stored at − 80 °C. The cells were randomly divided into three groups: control, UCB, and VX-765 groups. The astrocytes in the UCB group were stimulated in culture with 50 μM UCB in the presence of 100 μM human serum albumin at conditions mimicking those of hyperbilirubinemic newborns for the indicated times [7, 10, 27, 28]. In addition, cells in the VX-765 group were pretreated with 50 μM VX-765 for 1 h prior to incubation with the same concentration of UCB and human serum albumin. Additionally, the control group was treated with equal volumes of DMEM and human serum albumin.

#### MTT reduction

The modified indirect MTT method was performed to investigate cell viability under UCB stimulation, which could eliminate the interference of bilirubin deposits [29]. Briefly, astrocytes were cultured on 96-well plates. Following treatment, the supernatants were removed, and the cells were incubated with 200 μl of MTT (0.5 mg/ml) for 4 h in the dark at 37 °C. After incubation, the medium was discarded, and MTT formazan crystals were dissolved in 160 μl of isopropanol/HCl (0.04 M) with gentle shaking for 15 min at room temperature. The absorbance of each well was transferred to another 96-well plate. Then, the optical density (OD) of the dissolved formazan product was determined at 570 nm using a microplate. After background OD subtraction, the results were expressed as a percentage of the average control. Each experiment was performed in triplicate plates and repeated three times.

#### Lactate dehydrogenase assay

The release of LDH in the astrocyte culture supernatant was regarded as an indicator of the destruction of cell membrane integrity. The LDH cytotoxicity detection kit was used according to the manufacturer’s instructions. Treatment with LDH release reagent was used as a positive control to test the maximum LDH release. The absorbance was measured at 490 nm using a microplate reader. All readings were corrected for the potential interference of UCB absorption, and the results were expressed as a percentage of LDH release.

#### Trypan blue exclusion

Trypan blue exclusion was performed to analyze the loss of cell membrane integrity based on the principle that an intact cell membrane (a property of viable cells) is necessary for the exclusion of certain dyes. After treatment, both adherent cells harvested by trypsinization and non-adherent cells floating in the medium were collected. Subsequently, 4% trypan blue was added to the resuspended cells at a 1:10 dilution, followed by incubation for 3 min. The number of trypan blue-positive cells was counted by using a hemocytometer in triplicate and expressed as a percentage. The cytotoxic effect of bilirubin in the presence or absence of the caspase-1 inhibitor VX-765 was evaluated.

#### EtBr and EthD2 staining

Two different membrane-impermeant dyes, EtBr (molecular weight (MW) 394 Da) and EthD2 (MW 1293 Da), were used to examine the size of the membrane pore [30]. The astrocytes were plated onto poly-d-lysine-coated glass coverslips in 24-well plates. At the indicated time point, the adherent cells were washed with PBS, stained with either EtBr at 25 μg/ml or EthD2 at 25 μg/ml, and counterstained with the Hoechst 33342 according to the manufacturer’s instructions. The coverslips were analyzed using a fluorescence microscope (Nikon, Japan). The means and standard deviations were derived from counting a minimum of four random microscopic fields from two different coverslips per sample.

#### Western blot analysis

Total protein was extracted using a protein extraction kit with PMSF (1 mM), and the protein concentration was determined using the BCA assay. An equal amount of protein (50 μg) was loaded, separated by 12% SDS-PAGE, and transferred to PVDF membranes (Bio-Rad). The membranes were blocked with 5% non-fat milk (room temperature, 1 h), incubated overnight at 4 °C with a primary antibody (anti-caspase-1, anti-NLRP3, or anti-β-actin), and then incubated with an HRP-conjugated anti-rabbit secondary antibody (room temperature, 1 h). The protein bands were visualized with the G-BOX Imaging System (Syngene, Cambridge, UK) using an ECL assay kit (Bio-Rad).

#### ELISA

The levels of the IL-1β and IL-18 proteins in cultured supernatants were measured by ELISA assays according to the manufacturer’s instructions. The absorbance of the samples was measured using a microplate reader at an optical density of 450 nm. All samples were tested in triplicate.

#### TdT-mediated dUTP nick end labeling assay

Nuclear DNA fragmentation was detected by TdT-mediated dUTP nick end labeling (TUNEL) staining according to the manufacturer’s instructions. The cells were counterstained with DAPI and analyzed with fluorescence microscopy. TUNEL-positive nuclei emitted green fluorescence, whereas TUNEL-negative appeared blue. TUNEL-positive nuclei (green) were determined by randomly counting ten fields of the section, and the results are expressed as a percentage of the total nucleus population. The means and standard deviations were derived from counting a minimum of four random microscopic fields from two different coverslips per sample.

#### Statistical analysis

The statistical analyses were performed with SPSS, version 17.0. The data are presented as the means and SD. A normality test and a homogeneity test for variance were initially performed. If the data were in compliance with a normal distribution and homogeneity of variance, an ANOVA with Bonferroni’s post hoc test or a Student’s *t* test was performed; otherwise, a rank sum test was used. The categorical data were analyzed using the chi-square test. *p* < 0.05 was considered statistically significant.

## Results

### UCB induced caspase-1 activation in cultured rat cortical astrocytes

**Fig. 1:**
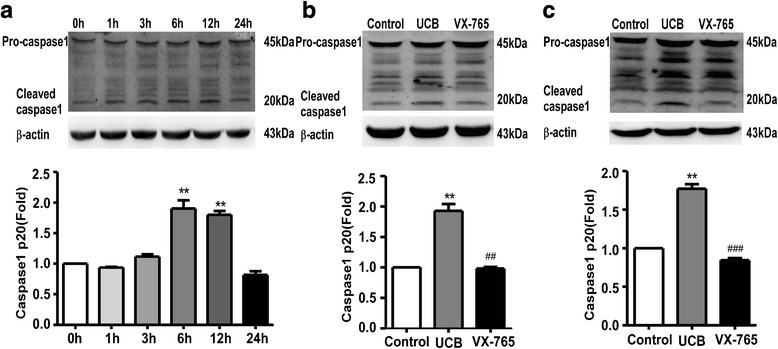
Caspase-1 activation in astrocytes treated with UCB. **a** Caspase-1 activity was measured by cleaved caspase-1 (p20) using western blotting at different time points after astrocyte treatment with UCB. **p < 0.01, versus the control group (0 h) using a two-tailed Student’s *t* test with Dunnett’s test. **b** VX-765 treatment inhibited the activation of caspase-1 at 6 h after UCB stimulation. **p < 0.01, versus the control group; ^##^*p* < 0.01, versus the UCB group using one-way ANOVA with Bonferroni’s post hoc test. **c** Caspase-1 activity was significantly attenuated in the VX-765 group at 12 h after UCB treatment. **p < 0.01, versus the control group; ^###^*p* < 0.001, versus the UCB group using one-way ANOVA with Bonferroni’s post hoc test. The intensity of the bands was quantitated by scanning densitometry, standardized with respect to β-actin protein, and normalized to the values of the control group. Three independent experiments were performed in duplicate. *Error bars*, SD

First, we used western blotting to investigate whether caspase-1 was activated in primary cultured astrocytes exposed to 50 μM UCB. The increased expression of the p20 subunit of caspase-1 (an activated form of caspase-1) was strongly detected at 6 h (*p* = 0.0026) and 12 h (*p* = 0.0022) and returned to baseline levels at 24 h compared with the control group (0 h) (Fig. 1a).

### VX-765 prevented caspase-1 activation of UCB-treated astrocytes

Subsequently, whether treatment with VX-765 could inhibit caspase-1 activation in the cultured cortical astrocytes upon UCB challenge was investigated. Based on the pharmacological characteristics of VX-765 and the peak time of caspase-1 activity in the astrocytes, VX-765 was administered at 1 h before UCB treatment. The activation of caspase-1 was examined by western blotting after incubating with UCB for 6 and 12 h, respectively. The caspase-1 activation at 6 h (*p* = 0.0021, Fig. 1b) and 12 h (*p* < 0.001, Fig. 1c) was significantly inhibited in the VX-765 group compared with the UCB group.

### Involvement of caspase-1 activation in UCB induced a loss of cell viability

**Fig. 2:**
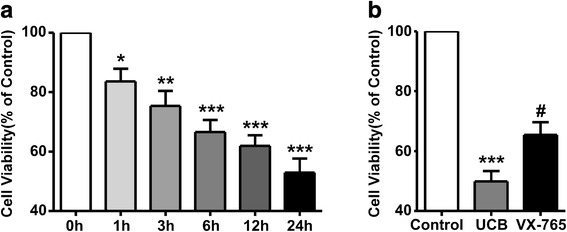
Involvement of caspase-1 activation in the UCB-induced loss of cell viability. Cell viability was determined with modified indirect MTT assay and presented as a percentage of the control. **a** The cell viability after treatment with 50 μM UCB was decreased in a time-dependent manner. *p < 0.05, versus the control group (0 h); **p < 0.01, versus the control group (0 h); ***p < 0.001, versus the control group (0 h) using the chi-square test. **b** The cells in different groups were treated as previously described for 24 h. ***p < 0.001, versus the control group; ^#^*p* < 0.05, versus the UCB group using the chi-square test. Four independent experiments were performed in duplicate

Modified indirect MTT assay was performed to assess the effect of VX-765 on the UCB-induced loss of cell viability in astrocytes. The results showed that cell viability declined in a time-dependent manner after exposure to UCB. Incubation with UCB markedly reduced the relative survival rate to 52.98 ± 11.52% of control values at 24 h (*p* = 0.0002, Fig. 2a). Next, the survival rate in each group was measured after UCB stimulation for 24 h. The survival rate of VX-765-treated astrocytes was 65.43 ± 10.34%, which was significantly higher than that of UCB-treated cells at 49.87 ± 8.63% (*p* = 0.0103, Fig. 2b).

### Caspase-1 activation is involved in UCB-induced plasma membrane rupture

**Fig. 3:**
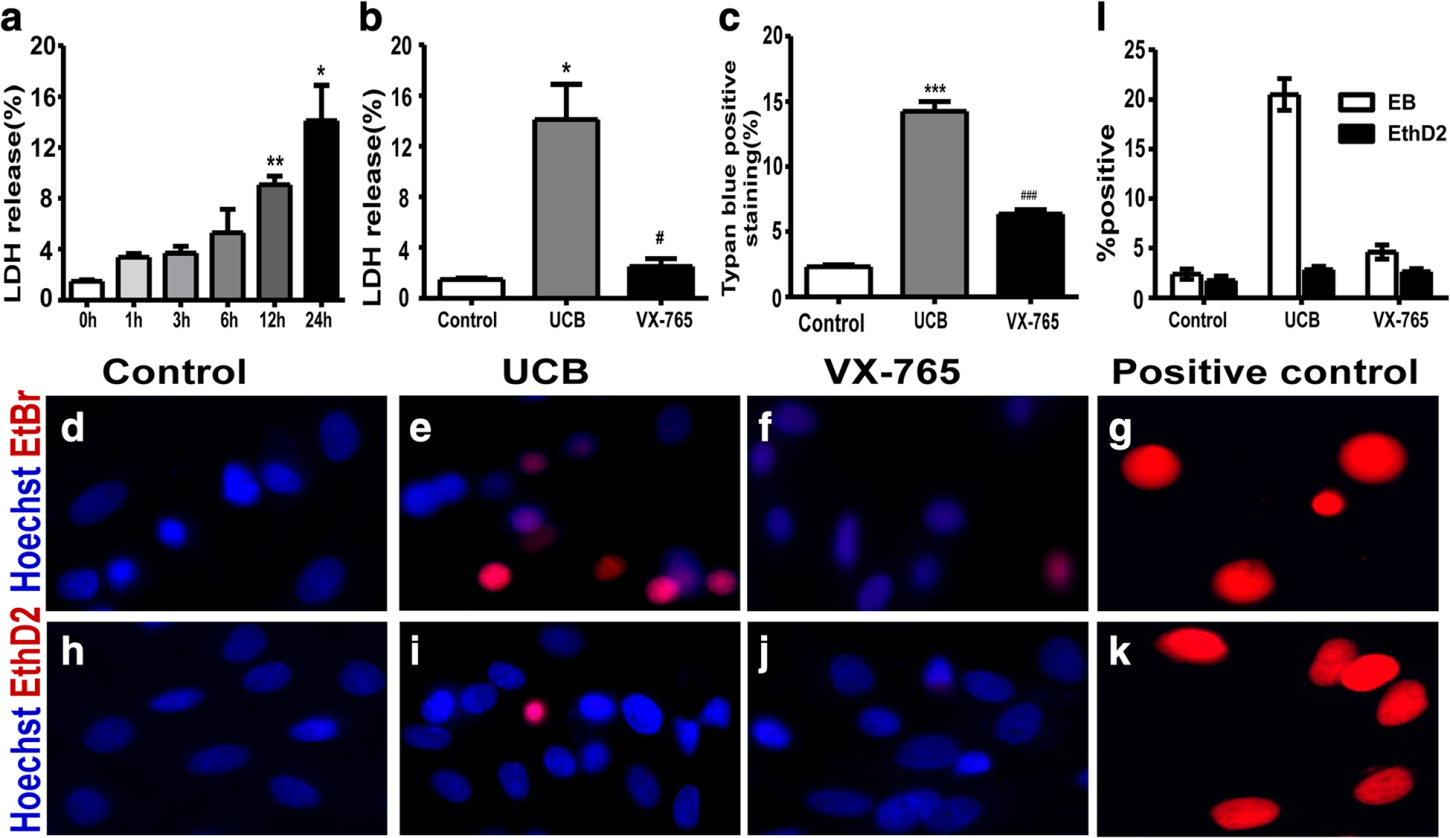
Caspase-1 activation involved in UCB-induced plasma membrane rupture. **a** Rat cortical astrocytes were treated as previously described for the indicated time periods. The supernatant was collected for the determination of released LDH activity. *p < 0.05, versus the control group (0 h); **p < 0.01, versus the control group (0 h) using a two-tailed Student’s *t* test with Dunnett’s test. **b** LDH released into the supernatant by a cell loss of membrane integrity at 24 h was quantified. *p < 0.05, versus the control group; ^#^*p* < 0.05, versus the UCB group using one-way ANOVA with Bonferroni’s post hoc test. Four independent experiments were performed in duplicate. **c** The cells positive of trypan blue staining in different groups were counted at 24 h after stimulation. ***p < 0.001, versus the VX-765 group; ^###^*p* < 0.001, versus the UCB group using one-way ANOVA with Bonferroni’s post hoc test. After treatment, the cells were stained with the membrane-permeable dye Hoechst 33342 (blue) and the membrane-impermeant dyes (red), EtBr (MW 394) or EthD2 (MW 1293). Adherent cells were visualized by fluorescence microscopy (× 40 objective). Representative images are shown. The cells in the control group excluded both EtBr (**d**) and EthD2 (**h**). As a positive control, cells treated with Triton X-100 caused the uptake of EtBr (**g**) and EthD2 (**k**). Cells cultured with UCB showed a higher influx of EtBr (**e**) and a smaller influx of EthD2 (**i**). The caspase-1 inhibitor, VX-765, prevented astrocytic EtBr uptake (**f**) but had no significant effect on EthD2 uptake (**j**). The positive cells (red) were determined by randomly counting a minimum of four fields from two different coverslips per sample and expressed as a percentage of the total nucleus population (**l**). The results were representative of five independent experiments. *Error bars*, SD

Then, the cell membrane integrity of astrocyte exposure to UCB was investigated. The release of LDH, a major component of the cytoplasm, was also examined to explore UCB-induced cell membrane disruption. The results showed that UCB induced membrane injury in astrocytes in a time-dependent manner, which peaked at 24 h (Fig. 3a). Next, the cells in different groups were treated as previously described for 24 h. The percentage of LDH release in the UCB-treated group was 14.13 ± 5.50%, which was significantly higher than that of the control group (*p* = 0.0194). Additionally, the percentage of trypan blue dye staining, an index of the loss of cell membrane integrity, was significantly higher in astrocytes incubated at 24 h with UCB compared to that in the control group (*p* < 0.001). In addition, the blockade of caspase-1 activity with VX-765 reduced LDH release from 14.13 ± 5.50 to 2.52 ± 1.24% (*p* = 0.0314, Fig. 3b) as well as the rate of trypan blue-positive cells (*p* < 0.001, Fig. 3c), which showed strong suppression in astrocyte rupture under UCB challenge.

Next, staining with small membrane-impermeant dyes was performed to further investigate the pore formation of astrocytes treated with UCB. Two red membrane-impermeant dyes with different MWs, including EtBr (MW 394 Da) and EthD2 (MW 1293 Da), were used. The cells were stained with EtBr or EthD2 and counterstained with the membrane-permeable dye Hoechst 33342 (blue). The cells in the control group were stained with Hoechst 33342 but excluded both impermeant dyes (Fig. 3d, h). In contrast, cells pretreated with Triton X-100, which could increase the permeability of the cell membrane, enabled the influx of EtBr and EthD2 (Fig. 3g, k). After 24 h of stimulation, a large number of EtBr-positive cells were observed in the UCB-treated group (Fig. 3e), whereas only a few cells were positively stained with the larger EthD2 dye (Fig. 3i). These findings indicated pore formation in astrocyte membranes, with diameters enabling the influx of small molecules but excluding larger molecules in UCB-treated astrocytes. Consistently, we observed that the inhibition of caspase-1 activation with VX-765 strongly decreased EtBr uptake from 18.72 ± 3.47 to 5.39 ± 1.88% under UCB challenge (*p* < 0.001, Fig. 3e, f). However, VX-765 treatment had little influence in EthD2 uptake (Fig. 3i, j). These results indicated that caspase-1 activation is involved in the loss of membrane integrity characteristics of EtBr uptake in astrocytes treated with UCB.

### Caspase-1 activation participated in UCB-induced DNA fragmentation

**Fig. 4:**
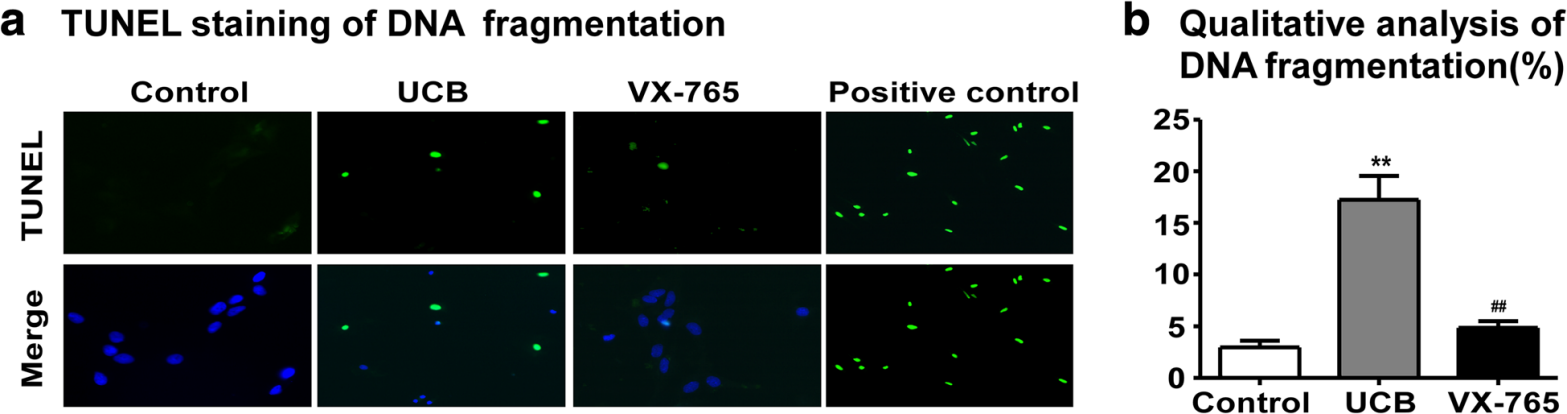
Caspase-1 activation participated in UCB-induced DNA fragmentation. **a** DNA fragmentation was measured by TUNEL staining (green). Adherent cells were visualized by fluorescence microscopy (× 40 objective). Representative images are shown. Fewer TUNEL-positive cells were observed in the VX-765 group than in the UCB group. **b** Qualitative analysis of DNA fragmentation was determined by randomly counting ten fields of the section and expressed as a percentage of the total nucleus population. The rate of DNA fragmentation was significantly reduced in the VX-765-treated astrocytes compared with the cells in the UCB group. **p < 0.01, versus the control group; ^##^*p* < 0.01, versus the UCB group using one-way ANOVA with Bonferroni’s post hoc test. Three independent experiments were performed in duplicate. *Error bars*, SD

TUNEL staining was performed to assess the DNA damage induced by UCB (Fig. 4a). Compared with the control group, a large number of TUNEL-positive cells were observed in the UCB-treated group (*p* = 0.0014, Fig. 4b). After treatment with a specific caspase-1 inhibitor, only a few cells were positively stained in the VX-765-pretreated group. The DNA fragment rates were calculated based on the average total number of cells and the number of TUNEL-positive cells in cultured rat cortical astrocytes in ten randomly selected fields. The results showed that the rate of DNA fragmentation was significantly reduced in the VX-765 group compared with the UCB group (*p* = 0.0026, Fig. 4b).

### Involvement of caspase-1 activation in UCB-induced cytokine release

**Fig. 5:**
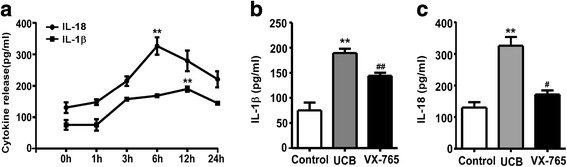
Involvement of caspase-1 activation in UCB-induced cytokine release. **a** The release of IL-1β and IL-18 at different time points in the cultured rat astrocytes. IL-1β and IL-18 secretion peaked at 12 and 6 h, respectively, after UCB administration. **p < 0.01, versus the control group (0 h) using a two-tailed Student’s *t* test with Dunnett’s test. The levels of IL-1β (**b**) and IL-18 (**c**) were dramatically decreased in the VX-765 group compared with the control group. **p < 0.01, versus the control group; ^#^*p* < 0.05, versus the UCB group; ^##^*p* < 0.01, versus the UCB group using one-way ANOVA with Bonferroni’s post hoc test. Three independent experiments were performed in duplicate. *Error bars*, SD

To evaluate whether caspase-1 activation was involved in UCB-induced cytokine release, the secretion of IL-1β and IL-18 cytokines was first detected in the culture supernatant of astrocytes. The cytokines were released in a time-dependent manner (Fig. 5a). IL-1β secretion began to increase at 3 h after UCB treatment, peaked at 12 h (*p* = 0.0035), and then decreased. IL-18 was upregulated at 3 h, peaked at 6 h (*p* = 0.0058) after model establishment, and then gradually declined. Therefore, we selected 12 and 6 h as the representative time points to measure the levels of these two cytokines and investigate whether inhibition of caspase-1 activity could reduce cytokine release in astrocytes under UCB challenge. The ELISA results showed that the secretion of IL-1β (12 h) and IL-18 (6 h) was significantly decreased in VX-765-pretreated astrocytes compared with the UCB group (*p* = 0.0187 and *p* = 0.0085, Fig. 5b, c).

### UCB induced the increased expression of NLRP3

**Fig. 6:**
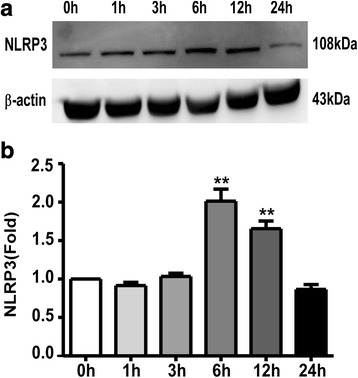
UCB induced the increased expression of NLRP3. Rat cortical astrocytes were treated as previously described for the indicated time periods. Total cell lysates were analyzed by western blotting. **a** Protein NLRP3 was measured by western blotting at different time points after astrocyte treatment with UCB. **b** The intensity of the bands was quantified by scanning densitometry, standardized with respect to β-actin protein, and normalized to the values of the control group (0 h). **p < 0.01, versus the control group (0 h) using a two-tailed Student’s *t* test with Dunnett’s test. Three independent experiments were performed in duplicate. *Error bars*, SD

A previous study demonstrated that the activation of caspase-1 was mediated by cytosolic multiprotein complexes, termed inflammasomes [31]. Increasing evidence has demonstrated that the NLRP3 inflammasome is associated with sterile inflammatory responses in various CNS disorders. Thus, the protein level of NLRP3 was assessed in total astrocyte lysates by western blotting. The results indicated that astrocytes treated with UCB exhibited obviously increased NLRP3 expression at 6 and 12 h (*p* = 0.0013 and *p* = 0.0012) compared with the control group (Fig. 6a, b).

## Discussion

Bilirubin-induced neurological dysfunction (BIND), a severe complication of neonatal hyperbilirubinemia, still occurs worldwide and leads to a high rate of mortality or lifelong neurological impairments [1, 2, 32, 33]. For years, research efforts have been made to understand how UCB induces neurotoxicity. Although remarkable advances were made, many challenges remained. The present study aimed to identify whether cultured rat cortical astrocytes undergo a novel form of cell death, pyroptosis, upon UCB challenge.

Notably, astrocytes, as the most abundant type of glial cells, are key mediators involved in the inflammatory responses in several CNS diseases [14]. Recently, several in vitro and in vivo studies have indicated that UCB-activated astrocytes release a variety of pro-inflammation cytokines, such as IL-1β and TNF-α, and eventually undergo time-dependent cell death [5, 7, 15–17, 34, 35]. Subsequently, these neuroinflammatory molecules released by active astrocytes induce recruitment and activation of additional glial cells secreting neuroinflammatory molecules, causing further cell damage [16]. Moreover, injury of astrocyte function had been closely associated with neurological sequelae [36]. Therefore, astrocytes were used in the present study.

To determine the central role of caspase-1 activation in pyroptosis [21, 23], western blotting was performed. The results showed that caspase-1 was time-dependently activated in cultured rat cortical astrocytes exposed to UCB, peaking at 6 h and returning to normal levels at 24 h. Caspase-1, as a member of intracellular cysteine proteases belonging to inflammatory caspases, plays a considerable role in the processing and secretion of pro-inflammatory cytokines, cleaving cytoplasmic proteins and cell survival in nervous system pathologies [21, 23, 37]. For example, the activation of caspase-1 promoted neuronal injury in experimental models of Alzheimer’s disease [24, 38, 39]. Additionally, caspase-1-deficient mice show resistance to hypoxic-ischemic developmental brain injury [40, 41]. However, the precise effect of caspase-1 activity on astrocytes under UCB challenge remains unknown. Therefore, an inhibitor was used to observe whether caspase-1 activation exacerbates the pathophysiology of this damage or contributes to repair mechanisms. In the present study, VX-765, a novel available prodrug of a potent and selective competitive inhibitor of caspase-1 was used. To date, this caspase-1 inhibitor is the first to enter clinic development for the treatment of inflammatory and autoimmune conditions without any signs of toxicity [37, 42]. Based on the pharmacokinetics of VX-765, the administration of VX-765 at 1 h prior to UCB treatment could prevent UCB-induced caspase-1 activation in astrocytes.

The present study indicated that UCB increased the release of LDH in a time-dependent manner, consistent with the results of previous reports [15], suggesting that the loss of cell membrane integrity was involved in UCB-induced astrocyte dysfunction [15, 16]. Notably, although the membrane defects in astrocytes treated with UCB have been well described, the details concerning pore formation in the plasma membrane remain unknown. According to a previous study, pyroptotic cells form plasma membrane pores between 1.1 and 2.4 nm in diameter [43, 44]. Thus, staining with small membrane-impermeant dyes of different molecular weights was conducted [30]. Interestingly, the results demonstrated that the UCB-treated astrocytes are permeable to small molecules, such as EtBr (MW 394 Da), while excluding the larger molecules, such as EthD2 (MW 1293 Da), based on the diameter of the membrane pore [30]. In addition, the EtBr uptake by astrocytes exposed to UCB was significantly reduced after treatment with VX-765, indicating that UCB could induce membrane pore formation with a functional diameter of 1.1–2.4 nm. Moreover, preventing EtBr uptake by the inhibition of caspase-1 activation suggested that pore formation was caspase-1 dependent [30]. Moreover, the data showed that VX-765 prevented UCB-induced LDH release and trypan blue staining. Taken together, these data demonstrated that the prevention of caspase-1 activation with VX-765 improved the survival rate of astrocytes under UCB challenge, partly owing to alleviating membrane damage.

Next, TUNEL staining was employed to investigate DNA fragment. The present study showed that UCB treatment obviously increased DNA cleavage in astrocyte at 24 h after model establishment. TUNEL staining is typically performed to observe cell apoptosis, considering that DNA fragmentation is a criterion of apoptosis [45]. However, recent abundant evidence suggests that the degradation of chromosomal DNA is also detected during pyroptosis, resulting in TUNEL-positive staining [43, 46]. Unfortunately, the enzyme directly responsible for DNA fragmentation during pyroptosis remains unknown [43]. Here, the experiment demonstrated that the blockade of caspase-1 activation with VX-765 apparently reduced TUNEL-positive astrocytes compared with that in the UCB-treated group. Thus, the DNA damage observed in the presence of UCB may, in part, be attributed to the activation of caspase-1. Additionally, the protective role of VX-765 in UCB-induced neurotoxicity might account for the decreased nuclear damage. Notably, previous studies have demonstrated that preventing caspase-3-related apoptosis could also attenuate UCB-induced DNA fragmentation [47]. Thus, it is therefore reasonable to speculate that both apoptosis and pyroptosis are involved in UCB-induced DNA fragmentation in astrocytes; however, additional studies are needed to determine which cell death process exerts a more important role.

Pyroptosis was characterized by not only rapid plasma membrane rupture but also the release of pro-inflammatory intracellular contents, particularly IL-1β and IL-18, attracting more cells to die [21, 42, 46]. Caspase-1 was activated during pyroptosis, initiating the release of mature IL-1β and IL-18 [21, 46]. Additionally, IL-1β induced a fast increase in extracellular glutamate contents, which affected the excitotoxicity of cells exposed to glutamate, leading to neural damage [48]. However, the pro-inflammatory cytokines activated by the MAPK signaling pathway and NF-κB signaling pathway via binding to IL-1 receptor 1 recruit more cytokines and immune cells, resulting in deleterious cell injury [5, 6, 20, 34, 35]. In the present study, IL-1β and IL-18 expression increased in UCB-treated astrocytes, whereas VX-765 could prevent this inflammatory cascade and inhibit the activation of caspase-1. Moreover, VX-765 alleviated the membrane damage of astrocytes, which might also prevent the release of cytokines. Consequently, it was proposed that VX-765 might reverse the bilirubin-induced neurotoxicity via suppressing pro-inflammatory cytokines and the subsequent inflammatory cascade, which may shed light on therapeutic targets against BIND.

According to the recent studies, the activation of caspase-1 is mediated by various inflammasomes [21, 49]. The present study focused on the NLRP3 inflammasome, the most extensively studied and clinically implicated inflammasome, which can be activated by a variety of dangerous signals, leading to sterile inflammatory responses involved in several neurological diseases [31, 50, 51]. Following the detection of stimulation signals, NLRP3 oligomerizes with ASC, resulting in autocatalytic cleavage and caspase-1 activation [51]. In the present study, UCB induced NLRP3 protein up-expression compared with that in the control group, which is in parallel with the activation of caspase-1. The increasing level of NLRP3 expression as well as the caspase-1 activity suggested that the NLRP3 inflammasome pathway may be involved in UCB-induced pyroptosis. However, the precise molecular mechanism by which the NLRP3 inflammasome is activated remains unknown. Therefore, additional studies are needed.

## Conclusions

In conclusion, the results of the present study suggest that the pyroptotic cell death modality is involved in UCB-induced neurotoxicity. Pyroptosis may play a vital role in the inflammation and development of BIND, which might be mediated by NLRP3 inflammation. More importantly, the prevention of caspase-1 activation, the core link of pyroptosis, reduced UCB-induced loss of cell viability and cytokine release, which might shed light on a new strategy to protect the developing brain against UCB neurotoxicity.

## Abbreviations

ANOVA: Analysis of variance
BIND: Bilirubin-induced neurological dysfunction
CNS: Central nervous system
DMEM: Dulbecco’s modified Eagle’s medium
DNA: Deoxyribonucleic acid
ELISA: Enzyme-linked immunosorbent assay
EtBr: Ethidium bromide
EthD2: Ethidium homodimer-2
FBS: Fetal bovine serum
GFAP: Glial fibrillary acidic protein
HSA: Human serum albumin
IL-18: Interleukin-18
IL-1β: Interleukin-1β
LDH: Lactate dehydrogenase
MTT: 3-(4,5-Dimethyl-2-thiazolyl)-2,5-diphenyl-2-*H*-tetrazolium bromide
NLRP3: NLR family pyrin domain containing 3
OD: Optical density
PMSF: Phenylmethylsulfonyl fluoride
SDS-PAGE: Sodium dodecyl sulfate polyacrylamide gel electrophoresis
TNF-α: Tumor necrosis factor alpha
TUNEL: TdT-mediated dUTP nick end labeling
UCB: Unconjugated bilirubin
